# ‘Having come to university my care was very much in my hands’: exploration of university students’ perceptions of health care needs and services using the common-sense model of self-regulation

**DOI:** 10.1007/s10865-020-00147-0

**Published:** 2020-03-26

**Authors:** Rayna Rogowsky, Anita Laidlaw, Gozde Ozakinci

**Affiliations:** School of Medicine, University of St Andrews, St Andrews, Fife, KY16 9TF Scotland, UK

**Keywords:** Symptom reporting, Health care use, Health care seeking, University students, Qualitative research

## Abstract

The health care needs and service experiences of higher education students require more research attention, given the increase in students who have a long-term illness, medical condition, or disability (“condition”). It is also important to consider the experiences of rising numbers of international students. This exploratory qualitative study used face-to-face interviews and the common-sense model of self-regulation to investigate students’ perceptions and coping behaviours, in a higher education institution in the UK. Thematic analysis was used to analyse the data. Twenty students who self-identified as having a condition were interviewed. This study adds depth to the understanding of the connections between students’ health-related experiences and their personal, academic, and post-graduation aspirations and the support needs of students, including international students. To optimise institutional support, innovations in partnerships with local care organisations and within the university, staff training about conditions, peer mentorship, and information outreach especially to international students, should be considered.

**Electronic supplementary material:**

The online version of this article (10.1007/s10865-020-00147-0) contains supplementary material, which is available to authorized users.

## Introduction

There are nearly 2.3 million higher education students in the UK (Universities UK, 2015) and increasingly there are students who live with a long-term illness, medical condition, or disability (Herts et al., 2014; Storrie et al., 2010). Within the higher education student population, the number of students living with various conditions is hard to quantify. The term “disability” may not resonate with some, leading to their exclusion from relevant statistics (Brooks et al., 2015; Royster & Marshall, 2008). The limited statistics available provide *some* insight into the number of students affected by a condition. According to UK-based Higher Education Statistics Agency (HESA), the percentage of full-time first-degree students from the UK receiving Disabled Student Allowances (DSA) has trended upward from 2000 to 2017; specifically, in Scotland, there was an increase from 1.1 to 4.6% (HESA, 2018). Only UK students are eligible for DSA, and some students who self-identify with a condition may not disclose it to higher education institutions which may mean that these numbers may underestimate the numbers of students affected (Royster & Marshall, 2008).

Living with a condition can impact various aspects of life over the course of the student experience, including academic outcomes such as increasing the likelihood of dropping out, disengaging from studies, and lowering grades (Eisenberg et al., 2009; O’Keefe, 2013; Storrie et al., 2010). Previous research using datasets such as from HESA provide a mixed picture regarding whether student disability predicts variation within student academic attainment (Richardson, 2009, 2010). Our qualitative study furthers the understanding of student experiences of conditions rather than focusing more narrowly on students registered with a disability, to elucidate associations with personal, academic and post-graduation aspirations and helps to inform the improvement of relevant institutional services (Royster & Marshall, 2008).

The health and wellbeing of students is identified as a top priority for universities (Audit Scotland, 2016; Lederer & Oswalt, 2017; O’Keefe, 2013). The UK legislation dictates that universities have the responsibility to protect students with disabilities, which could include those with mental or physical conditions (HM Government, 2013). The higher education student population has unique characteristics: students are managing stressful workloads, making independent decisions, and establishing new relationships, potentially impacting wellbeing regardless of whether or not a student has a condition (Laidlaw et al., 2016; O’Keefe, 2013). Neves and Hillman (2016) found in the Student Academic Experience Survey that undergraduate students in the UK have lower wellbeing measures compared to the general population and other young people. Students are potentially away from their home health care provider and health care system for the first time, which can be an especially significant transition for international students (Burgess (Munich) et al., 2016; Forbes-Mewett & Sawyer, 2016; Russell et al., 2008). There is limited research on the health care needs and service experiences of international students in the UK. In 2014–2015, 19.3% of all UK students came from countries outside of the UK (Universities UK, 2015). Thus, the factors impacting on international students’ health experiences, particularly those with significant health care needs, merit attention.

This study by using a qualitative and theory-driven method aims to contribute depth to the literature base about the experience of students living with conditions at university, highlighting the factor of home residence. Given the increasing number of higher education students in the UK living with conditions, understanding of their health care needs and service experiences to inform development and implementation of adequate support services to enhance student wellbeing requires more attention than in previous years (Herts et al., 2014; National Audit Office, 2007; O’Keefe, 2013; Storrie et al., 2010).

### Common-sense model of self-regulation

Leventhal’s common-sense model of self-regulation (CSM) is a theoretical framework for studying illness perceptions and management (Leventhal et al., 2012). According to the CSM, individuals form cognitive and emotional representations of the health threat. These cognitive representations have 5 dimensions: identity (what is the label for the threat and what are the experienced symptoms?), perceived cause (what is the cause of this threat?), perceived timeline (is it acute, cyclical, or chronic?), perceived control (expectations of control vs actual control), and perceived consequence of symptoms or a condition (its impact on daily life). These representations lead to the development of coping procedures for the treatment or the prevention of the health threat. The emotional representations of the health threats include fear and anger about the threat leading to either automatic (e.g., avoiding the issue) or deliberate attempts to control the distress (e.g., changing a health behaviour or seeking support). Individuals going through these processes then evaluate to what extent their coping behaviours achieved the desired outcomes. This model is appropriate for mental and physical health conditions and by using it as a lens to explore the illness perceptions and coping strategies of higher education students with conditions, we hope to gain a specific insight into their perceptions of their service needs and experiences which can inform the refinement of service provision.

### Aims

This exploratory qualitative study aimed to find answers to the following two questions:

1. What are the illness perceptions and related coping behaviours of students with conditions?
2. How does international status impact the illness perceptions and related coping behaviours of students with conditions?

## Methods

We used COREQ guidelines (Tong et al., 2007) and the SRQR checklist (supplement file) (O’Brien et al., 2014) to report our study:

### Study design

We used the CSM (Leventhal et al., 2012) and its conceptualisation of illness perceptions to gain a rich understanding of how students perceived their health care needs, and additionally, constructed meaning of their experiences of health services. For our purposes, a semi-structured interview approach towards data collection was considered most appropriate. Each interview began by asking the participant to fill out the Brief Illness Perception Questionnaire (BIPQ): an instrument for assessing illness perceptions containing eight questions (responses on a scale from 0 to 10), and a single open-ended question (Broadbent et al., 2006). The BIPQ was used as a guide for the semi-structured interviews; specifically, participants were asked to explain their answers to the BIPQ and describe any changes in their illness perceptions and self-management strategies from before arrival at university (or since first identifying with their condition) to their current situation. The research team developed the interview schedule informed by the BIPQ (see Results; see supplementary file for the interview guide). We analysed the transcripts using thematic analysis and applied illness perceptions as a framework. In other words, we coded the transcripts for the illness perceptions and perceptions and experiences related to international student status with openness to identifying additional themes not included in the CSM. RR conducted, audio-recorded, and transcribed all interviews. RR made notes at times following interviews as part of analysis process to help familiarise herself with the data and identify themes. We did not conduct repeat interviews nor return them to the participants for comment or corrections. Interviews lasted approximately 1 h with a minimum of 35 min and maximum 75 min.

We analysed the transcripts using thematic analysis (Braun & Clarke, 2006). Purposive sampling was employed for on-campus recruitment of participants who self-identified as having a condition. We advertised via student groups’ email lists and social media and by on-campus posters. Specific efforts were made to recruit international students via contact and posted advertisement through the international student support officer and international student-focused student groups. We conducted face-to-face interviews with participants who self-identified as having a “long-term illness, medical condition or disability” (hereafter referred to as “condition”). This terminology offered respondents a range of terms with which to identify and welcomed participants regardless of whether or not their conditions were diagnosed or reported to the university and could be interpreted as physical and mental health conditions. This recruitment strategy was selected as we wished to include anyone who may access healthcare or support services whilst at University (both psychological and physical) including those who had not declared themselves as disabled to the institution. Participants were offered £4 for their time and provided written informed consent. All interviews took place in a private room in the university. No one else was present during the interviews except the participants and the interviewer. Twenty participants completed interviews (see Table 1 for characteristics). No participants dropped out of the study. One individual attended for interview but did not consent or participate due to their misunderstanding of the eligibility criteria, as they did not self-identify as having a condition. We stopped recruitment at the point where we assessed we had reached information sufficiency. We created an ID for each participant (e.g., 1F): the number indicates the participant number and the letter indicates gender (F for female and M for male, GQ for Gender Queer). Seven participants reported more than one condition. Of these, three (4F, 1GQ, 14F) chose to answer interview questions for each condition, and four (3F, 2 M, 11F, 15F) did not choose to separate their conditions. Sixteen identified as female, three as male and one as gender queer, as indicated by Participant ID. The first 7 participants were undergraduates and informed that this was part of an MSc dissertation, which sampled only from undergraduate students. The other 13 participants were both undergraduates and postgraduates who were told that this was part of an expansion of the MSc project, which sampled from all students. Postgraduate student groups were targeted in the recruitment for the latter project. Apart from the efforts made to recruit international students, we did not seek specific characteristics among participants in the samples. Participants were asked to identify their race/ethnicity in their own words, but this information is not reported to ensure anonymity. Six identified home residences as non-UK and a 7th participant discussed her experience living overseas prior to university.

**Table 1:** Participant characteristics

ID	Condition
1F	Asthma
1M	Depression
2F	Polycystic ovary syndrome
3F	Complex regional pain syndrome, myalgic encephalopathy, tremor
4F	Dyslexia and anxiety^a^
2M	Depression, anxiety
5F	Short-sightedness
6F	Hashimoto’s disease
7F	Bulimia
8F	Myalgic encephalopathy
9F	Type 1 diabetes
3M	Purely obsessional obsessive compulsive disorder (Pure-0)
10F	Coeliac disease
11F	Major depressive disorder, generalized anxiety
1GQ	Chronic gastritis and anxiety/depression^^^
12F	Eating disorder
13F	Type 1 diabetes
14F	Arthritis and bipolar disorder (remission)^^^
15F	Eczema, acne
16F	Post Lyme disease syndrome

^a^Participant answered questions for each condition

### Research team and reflexivity

The female research team is comprised of a health professional (RR), behavioural scientist (AL), and health psychologist (GO). The interviewer (RR) was an MSc in Health Psychology student at the time of data collection with a nursing degree from a North American university who had previously worked in hospital-based nursing and community-based advocacy roles. She received training in conducting interviews and analysing qualitative data. She did not have a relationship established with the participants prior to the study. Stakeholders (Student Services) were consulted during the development of the research questions and the recruitment plan.

## Ethics

This study was approved by the School of Medicine, Teaching and Research Ethics Committee, University of St Andrews (MD12586).

## Results

RR initially deductively coded all transcripts for illness perceptions according to the CSM- cognitive (*identity, causes, control, timeline, consequences*) and emotional representations, as well as *perceptions related to international student status*. These codes were identified by the three data coders in advance. Following discussion with all authors, data related to coping strategies were incorporated into each theme and emotional representations were incorporated into the *perceived consequences* theme. These coding decisions were based on considerations of other qualitative studies applying the CSM (Hale et al., 2007; Heffernan et al., 2016; Webster et al., 2015). AL and GO independently reviewed transcripts and analysis notes.

## Cognitive representations

### Perceived identity

Identity perceptions involve both the labels that were given by health care professionals and the symptoms experienced by the participants. In our study, participants provided us with the label of the condition they self-identified with rather than using their medical records to confirm or determine what, if any, condition they reported to the university. We found that some participants’ identity perceptions conflicted with the explanations or labels provided by the health care professionals as well as expectations of others, including academic staff:

‘…Irritability, sleepless nights, this chronic cough which has pretty much been here for the last 4 months and last year I had the same thing which lasted 6 months, and I pretty much know it’s down to that [bulimia] but the doctors don’t seem to acknowledge anything.’ (7F, bulimia)

‘… it wasn’t like I missed class because I was in hospital because my blood sugar was really high, or I missed class because I collapsed because my blood sugar was so low, it was like I missed—I didn’t feel like I could say to a lecturer, oh you know I missed your class because my blood was high during the night and then I slept through my alarm in the morning? You know, they’re gonna be like, in my head they’re gonna be like you know you still slept through your alarm that’s not my issue, that’s not my problem,’ (9F, Type I diabetes)

For others, symptoms persisted even after they were deemed healthy and participants were frustrated that health care professionals did not understand this:

‘It’s almost like a red flag if you have Lyme disease, you don’t, there is not the same kind of respect that you get if you had say something like cancer which I think has a very clear, that you can say, this is an issue, we need to treat it. Whereas with Lyme disease, it’s hard to say, the doctors don’t often say, ‘this is major problem that we have to get to the bottom of’. (16F, post Lyme disease syndrome)

Identity perceptions that conflicted with what participants experienced from health services impacted coping behaviours. For example, 12F sought help from within the university as she believed she was not eligible for health service care based on her symptom experience:

‘I’m glad that student services you know will see me regardless of my weight.’ (12F, eating disorder).

The perceived expertise of a university-based mental health specialist helped 14F to manage anxiety about the future of her condition, particularly regarding fear of needing to leave university due to symptom recurrence. This, in turn, improved their coping with the condition. This also highlights how symptoms (“warning flags”) can interact with control beliefs (helpfulness of mental health specialist).

‘I found her quite helpful asking her some questions about that [symptom recurrence]. I find her more helpful than I previously found just the counsellors who maybe weren’t quite as prepared, yeah just thinking, maybe thinking even if it would be sort of sudden there should be some warning flags and stuff.’ (14F, bipolar disorder- remission).

### Perceived timeline

All participants experienced their conditions as being long-term. Their timeline perceptions were often defined by acceptance and hope:

‘I think I kind of take the view that there’s no point in being depressed about it because I’m gonna have it forever so I might as well just accept it.’ (13F, Type 1 diabetes).

‘I feel like it’s going to go on for quite a long time, even if I do it step by step. But even small steps are gonna help I think.’ (7F, bulimia).

Expectations of timeline were affected by information received from professionals:

‘…with the bipolar, something could trigger it and they’ve pretty much said it could it could, um, kind of come back at sort of any time, they’re just not sure.’ (14F, bipolar disorder- remission).

Perceptions of the future of their conditions interacted with the expected consequences of their conditions for many participants, including ability to plan for future as a student and beyond (See Table 2):

**Table 2:** Timeline perceptions related to future planning

(8F, myalgic encephalopathy	‘I’ve had to cater [for] it so that my day starts at 11 o’clock or 12 o’clock every day, and it makes me concerned for the future. Like how am I supposed to maintain a 9-5 job if I can’t guarantee attendance at 14 h of tutorial and class a week?’
(16F, post Lyme disease syndrome).	‘I feel very, very concerned sometimes, when I think what if this lasts for a long time, or for even longer, what does it look like if I want to have children, if I end up, the absolute last thing I would want is to be a mother who can’t, who can’t, be there for her children, and that would be, that would probably be my worst fear’
7F, bulimia	‘It kind of goes through phases, but I’m just concerned that things won’t get better or that they’ll continue to be a big factor in my life. Especially when I’m not at university when I won’t have so much control over when I work or what I do. Like, I’m going into medicine, I’m going to have to be on shifts and things like that where I can’t exercise as much as I’d like, and honestly that has that worries me a bit. But I think it’s step by step, eventually I’ll get there’

Timeline perceptions interacted with consequence perceptions related to participants’ personal and professional futures, which impacted coping at university, particularly their feeling ‘concerned’ whilst at university.

### Perceived causes

Participants’ perceptions of the causes of their conditions ranged from genetics, diet and medications, environment to luck and personality factors, and some distinguished between their own beliefs and those held by family members or professionals. For example, 9F described “very irrational, very religious-based” views of her family members as to why she has type 1 diabetes, whereas she said it is “a random thing that happened” and “it is autoimmune.” Some participants felt that aspects of university life worsened their conditions, and coping strategies at university were aimed to address these perceived causes. For example, 1F believed her asthma was exacerbated by student accommodation (e.g. dust), leading her to focus on improving her living conditions, such as acquiring “a really good hoover.”

At university, most participants experienced new levels of stress, which they believed worsened their health. 1F concluded that her asthma was more of a “joint problem” with mental health and based on a perception of psychological aspects to asthma, she pursued counselling at university. 12F believed the stress of transitioning to adulthood caused her eating disorder, as restricting eating “became a coping mechanism about the anxieties I was having about moving away and growing up.” This understanding led her to focus on behaviours such as doing her own food shopping and cooking at home to improve the transition back to university following a leave of absence.

### Perceived consequences

The beliefs about consequences at university were largely associated with academic and social experience as stated by 15F (eczema, acne):

‘…because it’s a condition that affects my mood obviously because it’s linked to self-confidence I think just um within university life whether it’s academic or socially it makes it harder to just go about your day to day stuff’.

Academic consequences included fluctuating involvement within learning sessions:

‘One tutorial I might be like super, you know, energetic and enthusiastic and then the next time I’ll be super low, you know, and sometimes it may appear that I’ve done the reading one week and I haven’t done the reading one week, but actually it’s I’m feeling great one week, and I haven’t taken my tablets the next week.’ (4F, anxiety).

For others, consequences of their conditions significantly affected their perceptions of their academic and career opportunities. Because only one laboratory was wheelchair accessible, 3F (complex regional pain syndrome) considered her dissertation options were limited: *‘*Everyone else got to pick their supervisor, I got told instead.’ 1F changed her course of study and career hopes due to consequence perceptions related to asthma:

‘I’ve actually changed my whole like dreams and career plans based on the fact that I have asthma, and that limits a lot what you can do. Because I was also, aside from wanting to be an explorer, I wanted to learn how to dive, and I wanted to fly for the RAF and all sorts of other things that I thought, oh I’d like to go do that. And I can’t do any of that because of it.’

8F who had coeliac disease was unsure about the potential for support to improve perceived academic consequences of her condition:

‘…it is making my studies harder but I’m not exactly sure what I could use. Like I’m sure I could use something but I don’t know what.’

Several participants suggested what helped and hindered coping related to academic consequence perceptions. Some participants identified that having a school-based ‘port of call’ for support was useful and discussed frustrations with inconsistency across schools. The differences in academic procedures across Schools “can make things a bit unpredictable,” according to 14F (*arthritis, bipolar disorder*-*remission*): ‘Each of the Schools has a disabilities officer, I don’t really see them getting used. I think if you’ve got a role like that they should be, you know, talking to the disabled students of the department just checking into see if they need anything specific to the department. Um I think I got an email once about one of my tutorial classrooms was upstairs but that’s about it.’

The perceived inconsistencies or confusion related to where to go for academic accommodation negatively impacted participants:

‘So I went and spoke to my tutor and I was like, “Obviously, I’ve missed a test but Student Services said they sorted it,” and they said, “You didn’t email us directly, so we’re not counting that, so you’ve got a 0 for that test, it’ll bring down your entire grade.”… missing 2 weeks of class isn’t great for anyone but I feel like because of the way it was dealt with, it’s really affected more than it has to. Um so that’s just a bit of a pain. Yeah but apart from that people have been really understanding, student services have been really helpful, my lecturers have been very accommodating.’ (8F, myalgic encephalopathy).

Several participants raised the topic of taking a leave of absence due to their experiences of their conditions as well as perceptions of others. In the eyes of several participants, leave of absence was perceived as a failure to manage or cope with their condition:

‘There was one point last year where they [Student Services] wanted me to take a leave of absence… And I was completely against it. Looking back, I probably could’ve done with a leave of absence (laughs), like, things weren’t great last year, but because it had been suggested to me, I felt like, “No, no, that’s admitting defeat. That’s people telling me that I can’t cope and that’s me admitting that they’re right, like I need to prove them wrong.’ (8F, myalgic encephalopathy).

The experience of taking leave of absence motivated 12F, as she did not want her condition to require her to leave university again.

‘I’m not going to let it affect my education, like I’m not going to let things slip so that I have to go home again.’ (12F, eating disorder).

For one of the participants who had taken a leave of absence because of her condition, consequences continued after she returned to study:

‘I experienced a relapse around this time last year, which caused me to have to take a leave of absence from the university. And so right now, I’m in a place where I’ve just restarted a year, so every day is sort of a reminder, because I’m taking the same courses as I did a year ago and all of my friends are now a year ahead of me, and I’m you know sort of estranged to them a little bit because you know I spent so much time away. That affects me quite a lot.’ (11F, major depressive disorder, generalised anxiety)

Most participants delayed seeking support until they perceived the consequences of their symptoms to be serious enough. 1F (asthma) interpreted her symptoms as “getting bad,” at university, which led her to seek local medical care. 2 M (depression, anxiety) only engaged with institutional services after academic progression was at-risk. He labelled his symptoms of depression as personal, and thus was reluctant to share with the university:

‘I felt like I was using the university. I’ve got respect for the university and I didn’t want to, you know, bring my personal life into it.’

Consequence perceptions relating to social experience focused on how others were perceived to react to their condition (See Table 3):

**Table 3:** Consequence perceptions related to social experience

2F, complex regional pain syndrome	‘I’m in real pain, and they’re like, ‘You’re imagining it, it’s fine’
12F, eating disorder	‘Students maybe do judge you because they don’t know, they don’t have experience with mental health…I thought I will tell my close friends that I made here because then if I’m having a bad day they’ll know what a bad day is.’ (12F, eating disorder)
14F bipolar disorder- remission, arthritis	‘There’s a lot more of a social stigma around it so it kind of affects me on a like, I feel a lot more comfortable telling people that I’ve got arthritis compared to like bipolar. And I think if it had been the other way around like I had arthritis in remission and bipolar not in remission then I think my life would in some ways be a lot harder’
15F, eczema, acne	‘The ideas around eczema are not the same as they are acne, like acne is something that happens in like kind of teenage years and it’s the sort of thing that you expect that can kind of go away. Having it as a young adult is like hard to deal with. So it’s kind of issues of self-esteem and self-confidence’

Some spoke about the potential benefits of peer networking opportunities within and/or external to the university:

‘I just wish there was a group where we could like talk about random things but know that we have something in common and know that we understand each other but not actively talk about like that we ate or what we haven’t ate. Just like having, like feeling that you are not alone.’ (12F, eating disorder).

‘I would want more support groups. We don’t do support groups here. The only support groups I found nearby even if I look as wide as (the county) as a whole, has been for sexual abuse, which is good, but that’s not for me (1M, Depression).

Participants deemed that an improved understanding among students and staff regarding the impact of having a condition at university could positively influence coping.

### Emotional representations

Emotional representations included feelings of stress, guilt, worry, and fears of being misunderstood or judged at university, especially due to stigma, service use, and absenteeism:

‘I kind of feel guilty and in some ways I’d almost rather there was like a liaison between the two [student and academic staff] rather than having to directly, because you, I don’t know, I guess you, I just worry a bit about their perception of me a bit.’ (14F, arthritis, bipolar disorder- remission). 2F perceived missing class as greatly consequential, causing fear. At times she found it difficult to reconcile this belief with the need for health care, and in the past chose to attend class rather than a doctor’s appointment. 1F believed that her asthma had the potential to lead to death, causing worry. She decided to report asthma as a disability on her postgraduate application, acknowledging its seriousness; however, she also suggested that asthma was not as serious as other conditions, which caused her embarrassment:

‘I have to say, so I’m applying to another degree now, and I actually, I felt embarrassed, you know when it says do you have any disabilities or things we should know about when you’re applying to university, and I felt embarrassed to say I have asthma… I feel embarrassed to say, because I feel like people don’t treat it as a particularly big deal or condition compared to people in a wheelchair or something like that.’

Emotional representations were also associated with perceived expectations for students:

‘…As a med student you get a bit worried like if I start going to the doctor for this that and the other thing does that affect my fitness to practice. And so I was quite reluctant to go at the beginning. Whereas now I feel that going to the doctor was the right thing, even if it did cause problems in med school, getting myself sorted is more important.’ (1GQ, anxiety).

The academic transition to third year for undergraduate students was identified as particularly stressful and related to expectations for students: ‘…by 3rd year the, I believe it would’ve been stress that triggered a serious relapse.’ (11F, major depressive disorder, generalised anxiety).

### Perceived control

Being a university student affected majority of the respondents’ perceptions of control with regard to managing their long-term conditions; in particular, the independence at the university offered access to professional services, social outlets, and a sense of responsibility for management of conditions. However, this was not the case for all:

‘I think I was doing it way better [at home] because I had someone to like to hold me who told me to take my medications, drink this, do this. But since I’ve come here, I’ve been like, because I’ve been living on my own for like 6 months now, and it’s my first time. I’ve never lived on my own. I could barely wash, do my dishes, or like wash clothes. So, um, because there’s no one who’s like constantly reminding me to have my medication I haven’t really been doing that. So, it’s been kind of different.’ (2F, polycystic ovary syndrome).

‘It’s been a jump from nothing. To something… when I moved to university, just having the services offered. It was more services offered here having access without parents’ involvement that made the difference; that allowed me to use them. Because even if I had all the university support services in the world, if I was still living at home I probably wouldn’t be able to use them.’ (1M, depression).

Disclosure to university improved confidence in management, as 12F said, knowing that “there’s someone like from the first day to kind of um touch base with and for someone to be there, if I do need the help” made a significant impact. 4F spoke to the beneficial interplay of disclosing to university and self-managing: ‘The support for it, is good [Student Services] and the Disability Student Allowance that I get, where I get kind of equipment like recording equipment… is wicked, but again, I think it’s just a thing you have to learn to live with, and deal with…’ (4F, dyslexia).

A sense of autonomy and peer support were significant aspects of control perceptions, contributing to them seeking medical and institutional support, or serving as primary source of support:

‘Having come to university my care was very much in my hands and uh as a result I have become better at saying ok yeah I guess I am not coping very well, I need help…granted some of that did come from friends saying, “You know, do you think maybe you should…?”’ (11F, major depressive disorder, generalised anxiety).

In terms of coping strategies that helped with the perceptions of control over their conditions, several participants discussed physical activity (See Table 4):

**Table 4:** Control perceptions related to physical activity

6F, Hashimoto’s disease	‘Yeah I think for me, doing sports that are offered, like it’s really, really helpful. I would put it as number one most helpful thing…’
14F, arthritis	‘…On an NHS level, you know like I know in some places they prescribe like exercises you know like gym sessions… but the ones [here] they don’t seem to do that. When I’ve sort of enquired about it I think sometimes the financial factors can be quite a deterrent for students, but I think it would end up saving the NHS more money in the long term
11F, major depressive disorder and generalised anxiety	‘I’m exercising so that’s probably the thing that makes me feel like I have the most control’

Those who identified as having conditions for a while were more confident regarding self-management of their condition and help-seeking:

‘I am feeling better at managing myself now, the more I’ve gone through it and the more I’ve had experience of it, the better I get at learning what’s good and what’s not good. Um, and knowing when to seek help.” (1GQ, anxiety).’

Overall, participants appreciated the services at university and local medical practices and had both positive and negative experiences influencing control perceptions. 1F recalled the impact of her doctor telling her, “Your asthma is not controlled enough” on her decision to accept a particular internship. This participant valued the local medical care and described registering with the local GP as “obvious,” compared to others who did not feel medical care was accessible. The waiting time and unavailability of mental health and other specialist services was significant for participants, and many were advised to seek private care. Coping was affected by perceptions related to private care. For 3M (Pure-O):

‘It’s the period of time that you have to wait, that’s just going to make health worse. So I’m happy in a sense that I seek help quickly, and I’m able to afford private medical care in this country, so I think it’s great.’

11F described feeling guilt about requiring services beyond what the institution could offer and yet she complimented the support available for other students whom she perceived as requiring less intervention: ‘Services that they offer were not intensive enough or rather not at the level I needed to make progress… I always recommend student services to everyone… I think that a lot of people can benefit from just having somebody to talk to.’

For another participant (7F) positive social support was the greatest factor in her perceptions related to control. She valued the counselling support she received at university but spoke emotionally about the support she felt she lacked from health services:

‘And I feel like the only reason I feel like I’ve been able to keep going is because I’ve got other people supporting me. Even if that’s not my GP. My friends and my family have been I think the biggest step in helping to overcome it, um, it would just be nice if the actual health care stuff saw it the same way.’

For 9F, the potential for more peer support factored into coping related to control perceptions: *‘*I almost wish I did know more people with Type 1 Diabetes because you know sometimes when I do get a bit upset when I feel like things are out of my control I feel like it would be really nice to talk to someone who understands that.’

Researching conditions (e.g., through internet searches, academic literature) was another means that several participants identified helped them with coping related to control perceptions (See Table 5).

**Table 5:** Research conditions: coping related to control perceptions

7F bulimia	‘I’ve done a lot of research into it, trying to help myself’
15F, eczema, acne	‘I was old enough to kind of like do my own research and figure out like what was the action I wanted to take for it’
12F, eating disorder	‘I’ll go and watch a YouTube video of someone who’s done recovery videos. And it’s like really motivational because it’s almost like Student Services is there but like out of hours because those videos are there you know like for support… I just like googled it like eating disorder recovery so there’s quite a lot of videos on there, they’re really helpful if you have like bad days’
3 M, Pure-O	‘I think I have read almost everything, I dare say, all the books in the world about Pure-O’

1 M had been waiting for psychiatric services for “about a year.” 2M (depression) expressed reluctance to identify with the word “control” because without medication he was “a mess.” He did not feel he had the medical support he required but emphasised the positive impact of practical support at university, such as a laptop loan.

‘Sometimes the practical help is just as good. Because anxiety, you may know this, it can be just realistic anxiety about something, you see… in my condition, it gets compounded, you see. So things like umm the laptop situation. You know, when I got it, that made my life so much easier.’

### Perceptions related to international status

An overseas participant had not yet coordinated local care with her home provider, and 10F (coeliac disease) “was hoping not to have to go to the GP here [at university]” but ultimately sought care after waiting “so long to make an appointment.” Another overseas participant 5F (short-sightedness) was resigned to keep her eye care at home, as she perceived the local treatment as “more expensive” and “less personal.” Non-UK participants felt they lacked knowledge about the health care system:

‘I think just like in general giving students more information about what’s available on campus is super important because they just come in and tell you that you have to register with a GP but they don’t really, especially for international students, we don’t really understand how the health care system here works, at all.’ (6F, Hashimoto’s disease).

Other participants understood the UK health care system but had expectations from their home health care experience which were not being met:

‘Since I’ve come here I’ve been trying to book an appointment and it’s always 3 weeks from the day that I want an appointment for, which doesn’t really help my case. Because if I’m sick now; and the thing is that my condition is not serious enough for them to treat it as an emergency, but I’m still sick, and I want to be seen maybe in the same week, and that does not happen here. And then that’s still a GP. And he’ll refer me to a gynaecologist. I don’t know how many weeks that’s going to take.’ (2F, polycystic ovary syndrome).

## Discussion

In this study, we explored the impact of the transition to higher education on the management of long-term conditions, with consideration of home residence, which is important in an increasingly international higher education environment. The CSM provided a theoretical lens for understanding the experiences of students with conditions. Topics identified, such as the disclosure of disability to a higher education institution, use of accommodation services for academic work, and pursuit of medical, institutional and social support, have implications for understanding the influences on student wellbeing as well as retention, attainment, and employability (Brimstone et al., 2007; Chew-Graham et al., 2003; Jung, 2003; Knis-Matthews et al., 2007; Knott & Taylor, 2014; Kranke et al., 2013; Storrie et al., 2010). Existing literature on the issues experienced by university students with conditions tends to be non-theory driven or quantitative in nature, using students presenting to institutions with disabilities (as defined within HESA datasets). This study adds depth to the understanding of the connections between students’ health-related experiences and their personal, academic, and post-graduation aspirations.

Participants self-identified a range of physical and mental health conditions, the majority being mental health conditions. Our qualitative study illuminates experiences of the students represented in recent large-scale reports of mental health-related concerns among university students. For example, the Institute for Public Policy Research (IPPR), found that the number of UK students who reported mental health conditions increased nearly fivefold from 2006 to 2016, and slightly less than half of students who reported conditions did not disclose to their university (Thorley, 2017). Further, NUS Scotland surveyed student support services at Scottish higher education institutions and found that 75% of institutions had an increase in utilisation over the course of one year (NUS Scotland, 2010). Evidently, institutions in Scotland face increased pressure to meet the mental health needs of students. The findings from this study suggested that some students might pursue mental health-related support while studying because they believe they have access to appropriate services for the first time and an increased sense of autonomy. Further, students might perceive that their conditions worsen over the course of their studies, leading them to support services. IPPR also found that the number of students leaving university due to mental health conditions increased 210% from 2009 to 2014 (Thorley, 2017). This, combined with the mixed reports of support seeking experiences from our participants, suggests that mental health of university students is an area where support services, perhaps for those seeking support for the first time, requires attention.

Although this study’s sample is small and focuses on a range of conditions and we cannot make claims about generalisability on the basis of research approach, the data suggest that an institutional approach to mental and physical health support that is integrated as much as possible would benefit students (to our knowledge, there is no record of how many HEIs provide an in-house mental and physical health care service). The findings from the Chronic Illness Initiative (CII), based at one university in the United States, offer a perspective relevant to the present study despite differing health care system contexts. The CII re-strategised support for students with conditions by addressing the “unpredictable waxing and waning course of illness” (p. 121) which can impact ability to complete coursework and attain employment, and requires different accommodations than typically offered by institutions (Royster & Marshall, 2008). The CII featured one-to-one peer mentorship, distance learning opportunities, support groups, and advocacy for financial issues. Symposia hosted by the CII that brought together stakeholders and advocates from both within and outside of the institution created further opportunities for students’ social inclusion on campus and connection to employment-related resources (Royster & Marshall, 2008). Academic support was the foundation of CII, and after 4 years demonstrated students participating in CII had comparable grades to those not participating, had greater on average retention rates after first year, and were more likely to return to university for their second academic year (Royster & Marshall, 2008).

The CII study offered an innovative example with components that could be effective in our study’s setting. In particular, raising awareness about the impact of conditions on academic life and including a wide range of stakeholders in support provision could be helpful. Career services is one particular university-based stakeholder that could be included in support provision as perceptions of conditions’ impact on future planning affected coping. The authors of the CII study did not address the issue of how to identify students with conditions (Royster & Marshall, 2008). Our study found that the messages from institutional and medical support providers significantly impact illness perceptions and related coping. The outreach to students at the start of university, as well as key times (e.g., exams and transition to 3^rd^ year) could include encouragement to those who self-identify with a condition to make themselves known to support services. More research is needed to assess potential approaches to monitoring the needs of such students (Eisenberg et al., 2007; Lemly et al., 2014). Several participants in this study wished university staff were more aware of their health care needs, a finding that fits with the work of the Higher Education Academy’s 2017 report on the topic of integrating mental wellbeing into curriculum and up-skilling academic staff (Houghton & Anderson, 2017). Ravert and colleagues recently found in a pilot study, that coping among students with conditions was most enhanced by increased access to knowledge about the effects of conditions on life at university and support, which align with the findings of the present study (Ravert et al., 2017). A recent report from Universities UK called for increased collaboration between NHS and universities regarding the mental health needs of students, which is a recommendation also supported by our findings (Universities UK, 2018). Participants in our study also spoke about the potential benefit of better links within the university, such as a ‘middle person’ within academic schools bridging support professionals and the lecturer/tutor.

Illness perceptions and the links between these perceptions (e.g., timeline perceptions and emotional representations) were identified within the participants’ transcripts and could be linked to coping behaviour described by participants such as taking leave of absence or looking for information and/or videos online. This suggests that the CSM could be a helpful theoretical framework to understand higher education student support seeking behaviour and inform service development (Leventhal et al., 2012). Consequence perceptions, specifically acute academic consequences like the topic of taking a leave of absence and seeking private care seemed particularly related to participants’ coping behaviours.

While there were many similarities between UK and non-UK students, some perceptions related to control and consequence were impacted by home residence. One consistent theme among all participants was the perceived long-wait times and inaccessibility of appointments. Short-waiting times has been identified in previous research as important for adolescent patients (Rutishauser et al., 2003). Previous research also concluded that due to the overwhelming amount of information that students, particularly international students, are given at the start of university, the on-going repetition of messages about services might be needed to increase professional service utilisation (Burgess (Munich) et al., 2016; Forbes-Mewett & Sawyer, 2016). Indeed, Li et al. (2018) found that 38.7% of 432 student participants surveyed at an Australian university suggested increased awareness raising of services would improve utilisation of mental health services (Li et al., 2018). Our study suggests that international students particularly require clear and repeated messages, perhaps this could be achieved by leveraging technological resources, about where and how to access health-related support.

### Limitations

Participants were all students at one institution, therefore generalisability may be limited. To gain a broader picture of all higher education students experiences more postgraduates, mature students, part-time students and students matriculated at multiple sites could be examined in the future. This study relied on self-reports from students and recruitment only took place on campus and there were a higher proportion of participants identifying as female. It is possible that a bigger or more diverse sample would have resulted if recruitment advertisements were posted in local medical care practices. Participants were invited to self-identify their conditions, and those with multiple conditions were welcome to either consider them separately within the interview or together. Those who opted to consider multiple conditions together or identified conditions that were not named by a medical provider may have given responses that differed conceptually from those who discussed an individual condition and conditions given by a professional that they accepted. Future research might ask participants explicitly if the condition they discuss is a medical diagnosis, and whether or not they reported conditions to the university and their reasoning. Also, further work should include distribution of genders to be able to consider whether or not the experience of students with conditions at university is affected by gender.

### Strengths

The CSM was an effective lens for qualitatively examining the experience of students with conditions, both physical and psychological (Leventhal et al., 2012). The self-reporting of conditions allowed for the inclusion of students who might not have chosen or been able to disclose a disability to the institution ensuring a broader perspective than may be achieved with a sample comprising only those students who disclosed their condition to the University. The number of international students and even distribution of students across academic years were additional strengths of this study.

## Conclusions

The findings from this theory-driven qualitative study have the potential to inform interventions aimed at supporting student wellbeing, including academic retention, attainment, and employability aspirations. Our participants self-identified with a range of mental and physical conditions that overlapped and that they needed to manage during their university years. Participants often reported a link between their needs for the management of their conditions and academic performance and how this at times caused a conflict. Some suggestions from the current study are: Increasing support for students to mitigate the effects of their conditions on their lives and academic performance through practical support (e.g. access to laptop loan, physical activity) and improving links with local medical care and within the university; engaging in an on-going outreach to students and staff about the impact of these conditions; and promoting peer support networking opportunities internal and external to the institution. In an increasingly international student population, this group warrants our attention for their health care needs and management of their conditions while they are in the UK.

## Electronic supplementary material

Below is the link to the electronic supplementary material.

Supplementary material 1 (PDF 348 kb)

Supplementary material 2 (DOCX 28 kb)

## References

[CR1] Audit Scotland. (2016). Audit of higher education in Scottish universities. Retrieved March 20, 2020, from http://www.audit-scotland.gov.uk/report/audit-of-higher-education-in-scottish-universities

[CR2] Braun V, Clarke V (2006). Using thematic analysis in psychology. Qualitative Research in Psychology.

[CR3] Brimstone R, Thistlethwaite JE, Quirk F (2007). Behaviour of medical students in seeking mental and physical health care: Exploration and comparison with psychology students. Medical Education.

[CR4] Broadbent E, Petrie KJ, Main J, Weinman J (2006). The brief illness perception questionnaire. Journal of Psychosomatic Research.

[CR5] Brooks F, Magnusson J, Klemera E, Chester K, Spencer N, Smeeton N (2015). HBSC England National Report 2014.

[CR6] Burgess K, McKenzie W, Fehr F (2016). International female students’ experiences of navigating the Canadian health care system in a small town setting. Intercultural Education.

[CR7] Chew-Graham CA, Rogers A, Yassin N (2003). “I wouldn’t want it on my CV or their records”: Medical students’ experiences of help-seeking for mental health problems. Medical Education.

[CR8] Eisenberg D, Golberstein E, Gollust SE, Eisenberg D, Golberstein E, Gollust SE (2007). Help-seeking and access to mental health care in a university student. Population.

[CR9] Eisenberg D, Golberstein E, Hunt JB (2009). Mental health and academic success in college. The B.E. Journal of Economic Analysis & Policy.

[CR10] Forbes-Mewett H, Sawyer A-M (2016). International students and mental health. Journal of International Students.

[CR11] Hale ED, Treharne GJ, Kitas GD (2007). The Common-Sense Model of self-regulation of health and illness: How can we use it to understand and respond to our patients’ needs?. Rheumatology.

[CR12] Heffernan E, Coulson NS, Henshaw H, Barry JG, Ferguson MA (2016). Understanding the psychosocial experiences of adults with mild-moderate hearing loss: An application of Leventhal’s self-regulatory model. International Journal of Audiology.

[CR13] Herts KL, Wallis E, Maslow G (2014). College freshmen with chronic illness: A comparison with healthy first-year students. Journal of College Student Development.

[CR14] HESA. (2018). Table C - Percentage of UK domiciled students in receipt of Disabled Students’ Allowance by location of HE provider and academic year: 200/01 to 2016/17. https://www.hesa.ac.uk/data-and-analysis/performance-indicators/widening-participation/table-c.

[CR15] HM Government. (2013). Equality Act 2010: Guidance. *Office for Disability Issues*, *2010*(1). https://assets.publishing.service.gov.uk/government/uploads/system/uploads/attachment_data/file/570382/Equality_Act_2010-disability_definition.pdf

[CR16] Houghton, A.-M., & Anderson, J. (2017). Embedding mental wellbeing in the curriculum: maximising success in higher education. Higher Education Academy. Retrieved March 20, 2020, from https://www.heacademy.ac.uk/knowledge-hub/embedding-mental-wellbeing-curriculum-maximising-success-higher-education

[CR17] Jung K (2003). Chronic illness and academic accommodation: Meeting disabled students’ “unique needs” and preserving the institutional order of the university. The Journal of Sociology & Social Welfare.

[CR18] Knis-Matthews L, Bokara J, DeMeo L, Lepore N, Mavus L (2007). The meaning of higher education for people diagnosed with a mental illness: Four students share their experiences. Psychiatric Rehabilitation Journal.

[CR19] Knott F, Taylor A (2014). Life at university with Asperger syndrome: A comparison of student and staff perspectives. International Journal of Inclusive Education.

[CR20] Kranke D, Jackson SE, Taylor DA, Anderson-Fye E, Floersch J (2013). College student disclosure of non-apparent disabilities to receive classroom accommodations. Journal of Postsecondary Education and Disability.

[CR21] Laidlaw A, McLellan J, Ozakinci G (2016). Understanding undergraduate student perceptions of mental health, mental well-being and help-seeking behaviour. Studies in Higher Education.

[CR22] Lederer AM, Oswalt SB (2017). The value of college health promotion: A critical population and setting for improving the public’s health. American Journal of Health Education.

[CR23] Lemly DC, Lawlor K, Kelemen S, Weitzman ER (2014). College health service capacity to support students with chronic medical conditions: A national survey. Journal of Adolescent Health.

[CR24] Leventhal H, Bodnar-Deren S, Breland JY, Hash-Converse J, Phillips LA, Leventhal EA, Camern LD, Baum A, Revenson TA, Singer J (2012). Modeling health and illness behavior: The approach of the commonsense model. Handbook of health psychology.

[CR25] Li W, Denson LA, Dorstyn DS (2018). Understanding Australian university students’ mental health help-seeking: An empirical and theoretical investigation. Australian Journal of Psychology.

[CR26] National Audit Office. (2007). Staying the course: The retention of students in higher education. Retrieved March 20, 2020, from https://www.nao.org.uk/report/staying-the-course-the-retention-of-students-in-higher-education/

[CR27] Neves, J., & Hillman, N. (2016). The student academic experience survey. *Higher Education Policy Institute*. Retrieved March 20, 2020, from http://www.hepi.ac.uk/files/1.Higher_Educational_Report.pdf

[CR28] NUS Scotland. (2010). Silently stressed A survey into student mental wellbeing. Retrieved March 20, 2020, from https://www.nus.org.uk/en/news/silently-stressed-report-reveals-soaring-mental-ill-health-rates/

[CR29] O’Brien BC, Harris IB, Beckman TJ, Reed DA, Cook DA (2014). Standards for reporting qualitative research: A synthesis of recommendations. Academic Medicine.

[CR30] O’Keefe P (2013). A sense of belonging: Improving student retention. College Student Journal.

[CR31] Ravert RD, Russell LT, O’Guin MB (2017). Managing chronic conditions in college: Findings from prompted health incidents diaries. Journal of American College Health.

[CR32] Richardson JTE (2009). The academic attainment of students with disabilities in UK higher education. Studies in Higher Education.

[CR33] Richardson JTE (2010). Course completion and attainment in disabled students taking courses with the Open University UK. Open Learning: The Journal of Open, Distance and e-Learning.

[CR34] Royster L, Marshall O (2008). The chronic illness initiative: Supporting college students with chronic illness needs at DePaul University. Journal of Postsecondary Education and Disability.

[CR35] Russell J, Thomson G, Rosenthal D (2008). International student use of university health and counselling services. Higher Education.

[CR36] Rutishauser C, Esslinger A, Bond L, Sennhauser FH (2003). Consultations with adolescents: The gap between their expectations and their experiences. Acta Paediatrica.

[CR37] Storrie K, Ahern K, Tuckett A (2010). A systematic review: Students with mental health problems-A growing problem. International Journal of Nursing Practice.

[CR38] Thorley, C. (2017). Not by degrees: Improving student mental health in the UK’s universities. IPPR. Retrieved March 20, 2020, from https://www.ippr.org/publications/not-by-degrees

[CR39] Tong A, Sainsbury P, Craig J (2007). Consolidated criteria for reporting qualitative research (COREQ): A 32-item checklist for interviews and focus groups. International Journal for Quality in Health Care.

[CR40] Universities UK. (2015). Patterns and trends in UK higher education.

[CR41] Universities UK. (2018). Minding our future. Retrieved March 20, 2020, from https://www.universitiesuk.ac.uk/minding-our-future.

[CR42] Webster R, Thompson AR, Norman P (2015). “Everything’s fine, so why does it happen?” A qualitative investigation of patients’ perceptions of noncardiac chest pain. Journal of Clinical Nursing.

