# Supplementary material for: ‘Having come to university my care was very much in my hands’: exploration of university students’ perceptions of health care needs and services using the common-sense model of self-regulation
Source: J Behav Med. 2020 Mar 26;43(6):943–55. doi: 10.1007/s10865-020-00147-0 (PMC7674182; doi:10.1007/s10865-020-00147-0)
Supplement: Supplementary file 1 — Supplementary material 1 (PDF 348 kb) [file 10865_2020_147_MOESM1_ESM.pdf]

Semi-Structured Interview

Instructions:
The following questions are aimed to understand your experience of living with your condition while at university, including your beliefs about your condition, as well as your perspective on self-managing, seeking and receiving care. There are no right or wrong answers to any questions. I am only interested in hearing about your thoughts and experiences. You have the right to skip any question you do not want to answer.

Demographic Information:
Your home residence (North America, South America, UK, Europe [non-UK], Africa, Middle East, Asia, Australia/Oceania): ________________
Home health care system (National Health Service e.g. UK, National Health Insurance, e.g. Canada, Social Health Insurance e.g. Germany, Private Health System e.g. USA, Etatist Social Health Insurance e.g. France, I Don't Know): __________________
Race/ethnicity: ________________
Gender: ________________
Year of Study: ________________
Are you 18-25 years old? (YES/NO) _____________________
Your long-term illness, disability, or medical condition (hereafter referred to as 'condition': ________________

Semi-structured interview questions:

Introduction
- Tell me when you were diagnosed with your condition?
- What has your experience with managing your condition been like prior to arriving to university?

Assessing Illness Perceptions
- Please describe your responses to each answer on the Brief Illness Perceptions Questionnaire regarding your condition
-

Assessing Changes in Illness Perceptions
- In thinking about your responses today, can you tell me how, if at all, your answers might have been different prior to arriving to university? We'll go through your responses one at a time.
- If you perceive you answers could have changed since arriving to university, what do you think has caused the changes?
- Do you think your condition affect your university life?

Self-management of Illness
- What has your experience been like managing your own health at university? How confident are you managing your health?
- What has affected (positively or negatively) your ability to self-manage your illness?

- What is self-management like being away from your home health care provider/system?
- How do you think your experience compares to that of your peers living with illness?
- Anything else to say about self-management?

Seeking care for Illness
- What has been your experience seeking care at university?
- What has affected (positively or negatively) your experience seeking care at university?
- How do you think your experience is affected by differences from your home health care provider/system? How satisfied are you with the continuity of care from your home health care provider/system? What could make this better?
- Anything else to say about seeking care?
- Since you came here, have you contacted your home care provider? Why?

Concluding Points
- What would be the ideal support in place for your health care while at university in the UK?
- Is there something we have not yet covered that you would like to discuss?
